# The Adhesion of Plasma Nanocoatings Controls the Shear Properties of GF/Polyester Composite

**DOI:** 10.3390/polym13040593

**Published:** 2021-02-16

**Authors:** Tomas Plichta, Veronika Sirjovova, Milan Zvonek, Gerhard Kalinka, Vladimir Cech

**Affiliations:** 1 Institute of Materials Chemistry, Faculty of Chemistry, Brno University of Technology, Purkynova 118, 61200 Brno, Czech Republic; plichta@isibrno.cz (T.P.); xcsirjovova@fch.vut.cz (V.S.); xczvonek@fch.vut.cz (M.Z.); 2 Institute of Scientific Instruments of the CAS, v. v. i., Kralovopolska 147, 61264 Brno, Czech Republic; 3 Department of Materials Engineering, BAM—Federal Institute for Materials Research and Testing, Unter den Eichen 87, D-12205 Berlin, Germany; Gerhard.Kalinka@bam.de

**Keywords:** plasma nanocoatings, glass fibre, polymer composite, short-beam strength, interfacial shear strength, work of adhesion, mechanical properties

## Abstract

High-performance fibre-reinforced polymer composites are important construction materials based not only on the specific properties of the reinforcing fibres and the flexible polymer matrix but also on the compatible properties of the composite interphase. First, oxygen-free (a-CSi:H) and oxygen-binding (a-CSiO:H) plasma nanocoatings of different mechanical and tribological properties were deposited on planar silicon dioxide substrates that closely mimic E-glass. The nanoscratch test was used to characterize the nanocoating adhesion expressed in terms of critical normal load and work of adhesion. Next, the same nanocoatings were deposited on E-glass fibres, which were used as reinforcements in the polyester composite to affect its interphase properties. The shear properties of the polymer composite were characterized by macro- and micromechanical tests, namely a short beam shear test to determine the short-beam strength and a single fibre push-out test to determine the interfacial shear strength. The results of the polymer composites showed a strong correlation between the short-beam strength and the interfacial shear strength, proving that both tests are sensitive to changes in fibre-matrix adhesion due to different surface modifications of glass fibres (GF). Finally, a strong correlation between the shear properties of the GF/polyester composite and the adhesion of the plasma nanocoating expressed through the work of adhesion was demonstrated. Thus, increasing the work of adhesion of plasma nanocoatings from 0.8 to 1.5 mJ·m^−2^ increased the short-beam strength from 23.1 to 45.2 MPa. The results confirmed that the work of adhesion is a more suitable parameter in characterising the level of nanocoating adhesion in comparison with the critical normal load.

## 1. Introduction

Controlled synthesis of functional coatings used for surface modified materials is a fundamental step in a wide range of applications. Plasma-enhanced chemical vapour deposition (PECVD) is one of the suitable methods that may be used to prepare plasma nanocoatings. With this method, it is possible to deposit tailored coatings of variable physical and chemical properties by simply changing the deposition conditions, such as the power delivered to the plasma discharge, the process pressure, the precursor flow or the addition of other working (reactive or inert) gases [1,2,3].

Polymer composites are high-performance materials that combine a flexible polymer matrix with reinforcing components (particles or fibres) with higher strength, elastic modulus and stiffness [4]; their outstanding properties are based on the synergism of both kinds of components. However, these components are mostly incompatible because their chemical and mechanical properties are quite different [5,6]. The typical elastic modulus is 70–80 GPa for glass fibres (GF) and 3.5–4 GPa for polyester matrix [7,8]. Incompatibility of composite components results in high shear stress at composite interfaces under mechanical and thermal loads. Thus, for composites under high loads, it is necessary to ensure a sufficient adhesion at the reinforcement–matrix interface in order to achieve efficient stress transfer from the matrix to the fibre through the interphase region [9]. Nowadays, various methods are commonly used to increase the adhesion of both particle [10] and fibre-filled [11] composites. When fibres are used, it is advantageous to modify their surface using a plasma nanocoating [12,13] or a gradient multilayer [14] instead of the often imperfect and nonuniform wet chemical process [14,15]. The tailored nanocoating, as a compatible interlayer, enables higher compatibility and formation of strong bonds between the fibres surface and the modified polyester matrix [5]. This leads to an increased adhesion at both the nanocoating–fibre and matrix–nanocoating interfaces and, thus, improves the properties of the resulting composite material [14,16,17]. The modification of the fibre surface by a gradient multilayer may be uniquely achieved by a plasma nanocoating prepared using PECVD with an organosilicon precursor, namely tetravinylsilane (TVS) [12,18,19]. Using this procedure, we obtained polymer composites reinforced with glass fibres with smooth gradients of mechanical and chemical properties between the composite components, mimicking natural and biological systems. The a-CSi:H and a-CSiO:H plasma nanocoatings prepared from TVS and its mixture with oxygen gas in various fractions were tested by nanoindentation and the nanoscratch test to determine their mechanical and tribological properties. However, since the observed critical normal load [20] as a measure of adhesion is affected by intrinsic and extrinsic parameters [21,22], the work of adhesion was determined instead [23,24,25].

In our previous study, we demonstrated the ability to increase the shear strength of a polymer composite by changing the deposition conditions used for the synthesis of plasma nanocoatings [18]. 

Experimental and model data [19] indicated a relationship between plasma nanocoating adhesion to glass fibre and the interfacial shear strength (IFSS) of a glass fibre-reinforced polymer composite, which was measured by a microindentation test [26]. It is therefore expected that adhesion of the nanocoating to the reinforcing fibre will be responsible for the shear properties of the polymer composite. In this study, we focused on the development of plasma nanocoatings with different adhesions to planar glass, measured by a nanoscratch test, to demonstrate its effect on fibre–matrix adhesion in a glass fibre-reinforced polyester (GF/polyester) composite using a short beam shear test [27,28]. Short beam shear [29] is a composite lamina method and was used to determine the short-beam strength for GF/polyester composites reinforced by plasma-coated GFs. The same composite samples were used to characterize the IFSS using a direct method, i.e., a micromechanical push-out test for individual fibres. The adhesion of plasma nanocoating to planar glass is compared with the short-beam strength and IFSS in a GF/polyester composite.

## 2. Materials and Methods

### 2.1. Plasma Nanocoating Deposition

The deposition of plasma nanocoatings was performed by means of PECVD employing a radiofrequency glow discharge. More details about the high vacuum chamber together with its schematic arrangement have already been published in Reference [30]. For this experiment, we used a batch reactor, which must be evacuated for each sample separately. This batch reactor was used to develop a tailored plasma nanocoating for a specific composite system, i.e., the fibre and the polymer matrix. The plasma nanocoating could be then used for the continuous surface modification of selected fibres using a roll-to-roll PECVD reactor suitable for mass production [30].

The tubular reactor used for this study provides an axially symmetrical plasma and was used to deposit plasma nanocoatings both on flat substrates and, above all, on long fibre bundles, by means of glass holders (flat substrates) or the glass frame (fibre bundles). In the case of the planar substrates, a silicon wafer was used with the dimensions of 10 × 10 × 0.6 mm^3^ from ON Semiconductor, Roznov pod Radhostem, Czech Republic (DSP, B doped, (100) orientation); the GFs were without commercial sizing (unsized) from Saint Gobain Adfors, Litomysl, Czech Republic (E-glass, mean fibre diameter 19 µm, 1200 tex and 1600 fibres per bundle).

Tetravinylsilane (TVS, 97% purity, Sigma Aldrich, Prague, Czech Republic) was used as an organosilicon precursor for plasma polymerisations. Oxygen gas (99.99% purity, Linde Gas, Brno, Czech Republic) was used both for pre-treatments and in a mixture with TVS for deposition of the plasma nanocoatings as such. Two sets of six samples were prepared at power of 2 and 30 W, which was expected to vary the nanocoating adhesion. A power of 30 W was supplied in a continuous wave, while 2 W samples were deposited using pulsed plasma (total power 10 W, 20% duty cycle) due to the instability of the plasma in a continuous wave at low power levels. Various concentrations of oxygen in the mixtures with TVS were used for each set; the TVS flow rate was constant at 4.0 sccm, and the oxygen flow rate increased as follows: 0 sccm (0%), 2.0 sccm (33%), 2.9 sccm (42%), 4.3 sccm (52%), 6.2 sccm (61%) and 10.0 sccm (71%). These nanocoatings were prepared in two thicknesses, verified by mechanical profilometry (Dektak XT, Bruker, Billerica, MA, USA), for the nanoscratch (NS) and nanoindentation (NI) tests described below. The main parameters related to the preparation of the plasma nanocoating are captured in Table 1; more details and individual steps of the deposition are described in Reference [18].

### 2.2. Mechanical and Adhesion Testing of the Plasma Nanocoating

The plasma nanocoatings on the planar silicon wafer were tested using a 2 D TriboScope-75 (Hysitron, Minneapolis, MN, USA) attached to a modular Scanning Probe Microscope NTEGRA Prima (NT-MDT, Moscow, Russia) in order to obtain information on the plasma nanocoating adhesion to the substrate as well as the mechanical and tribological properties.

Nanoscratch testing of the 0.1 µm thick a-CSi:H/a-CSiO:H plasma nanocoatings was performed using a conical diamond tip (the radius of curvature 1.1 µm). During the test, a normal load was linearly increased during the test from 2 µN to 6 mN on a 10.0 μm scratch track over a time period of 30 s. Based on the measurements, the critical normal load (*L*_c_) was determined as the measure of plasma nanocoating adhesion to the substrate. This has been discussed before in [20], which also gives further details of measurement and evaluation. The average values and standard deviations were calculated from ten measurements.

A similar test was used to evaluate the friction coefficient. However, in this case, the indenter tip permeates through the nanocoating at a constant load of 750 µN. The friction coefficient was then calculated as the ratio of the lateral to the normal force in the constant measurement range [31].

The elastic modulus was studied through nanoindentation of the plasma nanocoatings, which were 1.0 μm thick. The test was performed by a Berkovich tip (50 nm tip radius) using cyclic nanoindentation up to a peak force of 10 mN in 300 s. The obtained 20 cycles were analysed by the Oliver and Pharr method [32] and yielded a depth profile of the elastic modulus. It was subsequently extrapolated to zero contact depth in order to obtain correct values without substrate influence. Five measurements were averaged, and the standard deviation was determined. More details about this method are found in [20,33].

### 2.3. Short Beam Shear Testing

The plasma nanocoatings were deposited on a GF bundle without sizing. It is necessary to consider the fact that the nanocoating deposited on the fibres inside the bundle are thinner than on the surface of the bundle (by an order of magnitude) because of the existence of a shielding effect [34]. However, all the fibres need to be surface-coated. Therefore, the thickness of the plasma nanocoating on the surface of the bundle at the location of the slowest nanocoating growth of 0.2 µm was chosen considering the results of the previous experiments [18].

The unsaturated isophthalic polyester resin (POLY DS 183 B1, Skolil Kompozit spol.s r.o., Prague, Czech Republic) together with additives (crosslinking agent, initiators, UV absorber and fibre wettability additive) was dosed into a mould. The plasma-nanocoated fibre bundle was then placed in the mould with the resin and thoroughly impregnated. During the preparation, particular attention was given to ensure that all the fibres had the correct uniaxial orientation, avoiding fibre crossing. Thus, a total of 24 bundles of fibres were placed, and the polyester matrix was added or removed as needed. Subsequently, the sample of the composite profile was cured; cut to the required size of beams, which were finely ground on a metallographic wet grinder; and then dried. The volume fraction of fibres in the composite samples prepared this way was 37%.

A macromechanical testing method—the short beam shear test (SBST)—was used to determine the short-beam strength of the composite beam [35,36,37]. This method is a three-point bending test, but due to a very small span of the supports, the effect of compressive stress is limited. The magnitude of the force is then directly proportional to the shear stress [4]. Short beams with the dimensions 18 × 10 × 3 mm^3^ were tested with respect to the standard (ASTM—D 2344/D 2344M) [29] by a universal testing machine Zwick Z010/TH2A (Zwick GmbH & Co, Ulm, Germany). The obtained values of the maximum load *P*_max_ were used to calculate the short-beam strength *τ*_int_ according to the following relationship given by the standard [29]:

(1) $$\tau_{\mathrm{int}}=\frac{3P_{\max}}{4tb}$$

where *t* is the beam thickness, and *b* corresponds to the width of beam.

### 2.4. Single Fibre Push-Out Testing

A slice of a composite beam was embedded in a PMMA tube (fibre in the same direction as the tube). Then, one side of this cylinder was grounded and polished using SiC sandpaper to a final grit size of 4000 (Struers Inc., Cleveland, OH, USA). From the polished side, a disk with a thickness of approximately 250–300 µm was cut from the cylinder with a diamond saw blade (Leica SP1600, Leica Biosystems, Nussloch, Germany). Afterwards, the disk was glued to a sample holder and the second side was polished using a micro-grinder Exakt 400 CS (EXAKT Advanced Technologies GmbH, Norderstedt, Germany). Online thickness monitoring was used to ensure a final thickness of the disk of about 30–35 µm. While polishing, the samples were lubricated and cooled by water.

With the push-out test [38], a single fibre was mechanically pushed out of the composite material by a flat punch indenter [36,37]. The scheme, test equipment, and experimental and model data were described in detail by Kalinka et al. [39]. A flat punch sapphire tip with a diameter of 14.5 µm was chosen for these measurements. This ensured an axial stress transfer into the fibres, which have diameters from 16 to 21 µm. Furthermore, this tip helped to prevent the contact of the polymer matrix when loading. The pushing speed was constant 0.4 µm·s^−1^. The sample was supported by a grid that provided free space below the tested fibre. The complete sample holder could be moved in two positions: under a microscope and under the push-out indenter. First, in the position under the microscope, a suitable fibre was selected. This should not have any damage, visible delamination, or contact with an adjacent fibre. Then, the sample holder was moved to the position directly under the indenter and the fibre was slowly pushed out of the disk. The test was terminated when the fibre completely de-bonded from the matrix and only sliding occurred. The individual phases of the test and the corresponding parts of the load–displacement curve of the push-out test were described in References [36,40]. The assumption in the subsequent analysis is that the shear stress is constant along the entire length of the fibre, and thus, the average shear strength is equal to

(2) $$\tau_{s}=\frac{P}{2\pi rh}$$

where *P* is the peak load; *r* is the diameter of the given fibre, which is determined using the optical microscope; and *h* corresponds to the fibre length, which is the thickness of the tested disk. The disk thickness was determined after a test series by using a Confocal Laser Scanning Microscope (VK-X100, Keyence, Osaka, Japan). The peak load arising from the test was substituted into Equation (2), and the interfacial shear strength (IFSS) was calculated [36]. Identifying the interfaces around the fibre and investigation the composite interphase are challenging topics. These topics were dealt with in detail in our previous papers [5,34], where atomic force microscopy (surface topography, phase imaging and lateral forces), atomic force acoustic microscopy and dynamic mechanical analysis (modulus mapping) were used for characterization of the interphase region of unsized, industrially sized and plasma-coated glass fibres in GF/polyester composite.

## 3. Results and Discussion

### 3.1. Adhesion of Plasma Nanocoatings in Terms of Critical Normal Load

Two sets of plasma nanocoatings deposited on silicon wafers were prepared from a TVS precursor in a mixture with oxygen gas at different concentrations (0, 33, 42, 52, 62 and 71% O_2_ in TVS/O_2_ mixture) because it has previously been shown that the nanocoatings prepared from TVS/O_2_ mixture improved the shear properties of a polymer composite in contrast to plasma nanocoatings prepared from pure TVS [41]. Since there is a 3 nm thick layer of native SiO_2_ on the surface of the silicon wafer, adhesion of the plasma nanocoating to this substrate can be considered identical to the glass substrate [19] and to GF, as has already been demonstrated [42]. Indeed, it has been found that, for a given film, the nanoscratch test leads to consistent nanocoating adhesion for both fibrous and planar glass substrates [42]. Plasma nanocoatings were subjected to a nanoscratch test to characterize the nanocoating adhesion.

**Figure 1:**
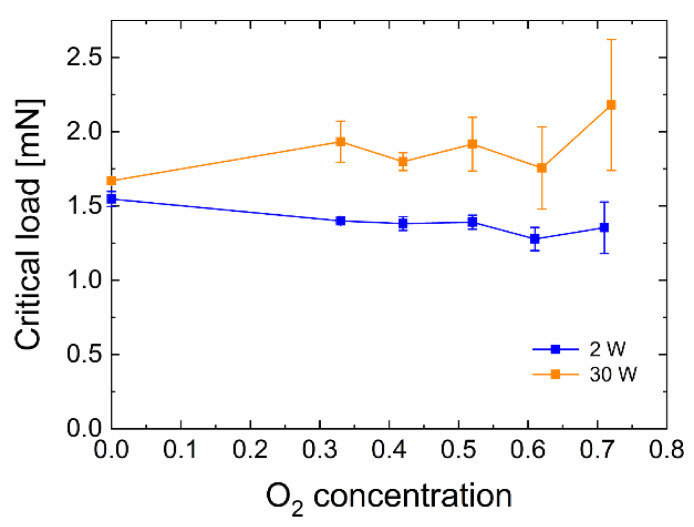
Dependence of the critical normal load vs. oxygen concentration for samples prepared at two different powers (2 and 30 W).

The displayed values of the critical normal load as results of the scratch test represent the measure of plasma nanocoating adhesion to the substrate (Figure 1). The critical normal loads for the 30 W samples (1.7–2.2 mN) are higher than that for the 2 W samples (1.5–1.3 mN). The distribution trend for a given set of samples is insignificant due to some higher standard deviations. A standard deviation for this type of test is generally around 10% [43,44]. Based on previous results [19,41], the higher adhesion of plasma nanocoatings deposited on GFs at 30 W should be reflected in the higher short-beam strength of the GF/polyester composite compared to nanocoatings deposited at 2 W.

### 3.2. Short-Beam Strength of GF/Polyester Composite

**Figure 2:**
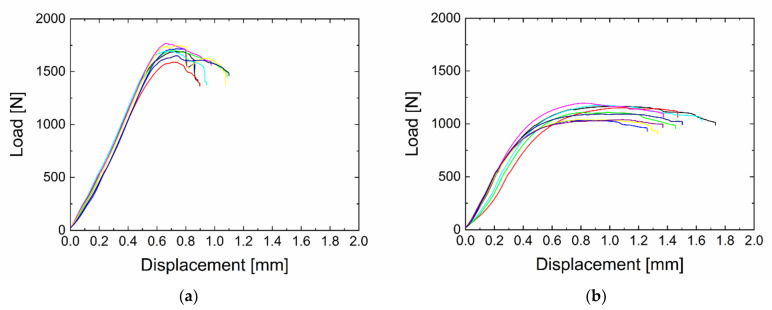
Load vs. displacement curves obtained by the short beam shear test (SBST) for a glass fibre (GF)/polyester composite with plasma-coated GFs at 33% oxygen in the tetravinylsilane (TVS)/O_2_ mixture and at a power of (**a**) 2 W and (**b**) 30 W.

Typical load–displacement curves resulting from the short beam shear testing of GF/polyester composite beams are shown in Figure 2 for plasma-coated GFs at 2 and 30 W and 33% oxygen in the TVS/O_2_ mixture. In the first case, it is evident that the maximum load reaches higher values, and thus, the adhesion of the fibres to the matrix is higher than in the second case. The reason for this is that a higher adhesion results in more efficient transfer of stress from the matrix to the fibres; this leads to an increase in the maximum load required for failure of the composite sample, resulting in higher values of the short-beam strength. For the sample with the higher adhesion (2 W), a sharper decrease in load after reaching its maximum is evident as well. This is also caused by more efficient stress transfer when individual fibres in one plane fail at the same time, followed by a sharp load decrease. On the other hand, for the 30 W plasma nanocoatings, the individual fibres gradually fail and ultimately causes a gradual decrease in load.

**Figure 3:**
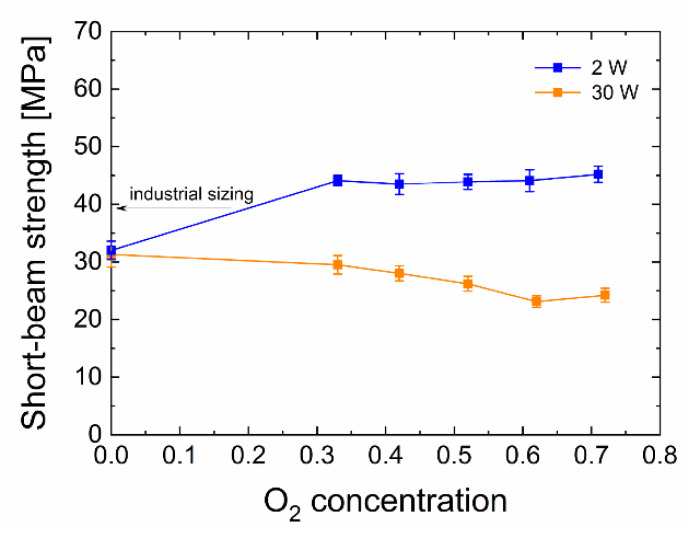
The relationship between short-beam strength and oxygen concentration in the TVS/O_2_ mixture for two different powers. The arrow indicates the short-beam strength value of the GF/polyester composite with industrially sized glass fibres.

According to Equation (1) and using the maximum load and size parameters of short beams, the resulting mean values of short-beam strength were determined together with standard deviations, always for 8–10 measurements. Figure 3 summarises the short-beam strength as a function of oxygen concentration in the TVS/O_2_ mixture for the two sets of samples (2 and 30 W). The short-beam strengths for unsized and industrially sized GFs are 13.8 MPa and 39.2 MPa, respectively [18].

For the composite samples prepared from fibres with plasma nanocoating that were deposited from the pure monomer using 2 W, it is clear that the shear strength reaches a similar value as that with the fibres using 30 W a-CSi:H plasma nanocoating, i.e., 32.0 MPa and 31.3 MPa, respectively. For the TVS/O_2_ mixtures with 33–71% oxygen, the set of samples prepared at 2 W shows an increase in short-beam strength in comparison to the interlayer of pure TVS; these values can be found at the level of 44–45 MPa. Based on previous results [19,41], we expect that this increase over the oxygen-free interlayer is caused by an increased number of covalent bonds between the GF surface and the plasma nanocoating due to the higher presence of Si–O–Si and Si–O–C groups, as proven by FTIR spectra [18]. In the 30 W set of samples, the shear strength decreases gradually from 31.3 MPa (0% oxygen) with increased oxygen fraction in the TVS/O_2_ mixture to 24.2 MPa (71% oxygen). This decrease may be explained by the fact that more C=O groups formed in the nanocoating material, thus reducing the number of covalent bonds between the plasma nanocoating and the polymer matrix [19,41] as an undesirable side effect. Moreover, plasma nanocoatings deposited using lower power (2 W) contain a larger amount of vinyl groups, which are responsible for the formation of covalent bonds between plasma nanocoating surface and the polyester resin during the curing process. This results in a higher strength of the interlayer/matrix interface [45]. In Figure 3, the arrow points to the short-beam strength corresponding to the commercial sizing of GFs, which is 39.2 MPa, and thus 13% lower than plasma-coated GFs at 33–71% oxygen and 2 W. However, the idea in Section 3.1. that a higher adhesion of plasma nanocoatings will result in higher short-beam strength has not been confirmed; therefore, analysis of the shear properties of the composite were continued using a direct method for individual GFs.

### 3.3. Interfacial Shear Strength in GF/Polyester Composite

**Figure 4:**
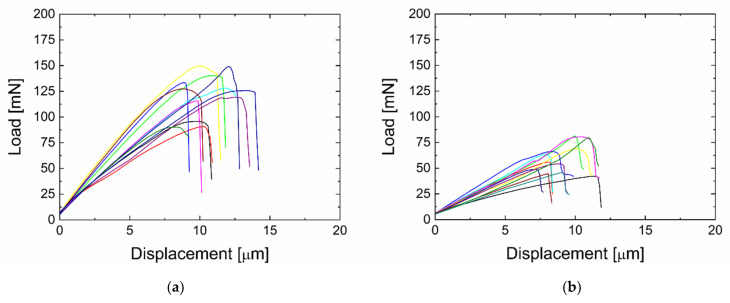
Load–displacement curves obtained by the push-out test for composites with plasma-nanocoated GFs using 33% O_2_ in the TVS/O_2_ mixture and at a power of (**a**) 2 W and (**b**) 30 W.

Thin disks were prepared from the same composite beams, which were also analysed by SBST, as described before. It was an identical polymer composite reinforced with surface-coated a-CSi:H/a-CSiO:H GFs prepared at 2 or 30 W. Push-out tests were used to determine the interfacial shear strength (IFSS) from load–displacement curves, which are shown for the two composite samples in Figure 4. Thirty measurements were made on each sample; twelve of them are shown, each with a different colour (Figure 4). The loading curves of the individual tests are again for the a-CSiO:H plasma nanocoating prepared from the mixture of TVS with 33% oxygen for 2 W and 30 W. The 2 W plasma nanocoating reaches higher values of maximum forces, approximately twice as high. As with SBST, the composite with this plasma nanocoating indicates a higher adhesion between the GF and the polymer matrix. This is evident from the higher slope of the loading curves. From the obtained loading curves, the values of the maximum load at which the interphase broke were determined. However, the interface between the fibre and the plasma nanocoating as an interlayer or the interface between this interlayer and the modified matrix may be broken in various ways to a different extent.

**Figure 5:**
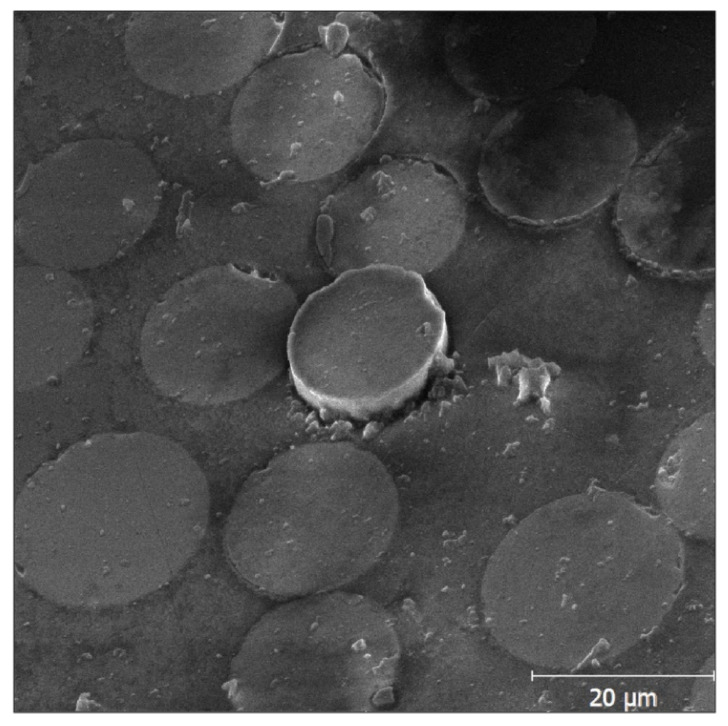
SEM image of the pushed-out fibre.

**Figure 6:**
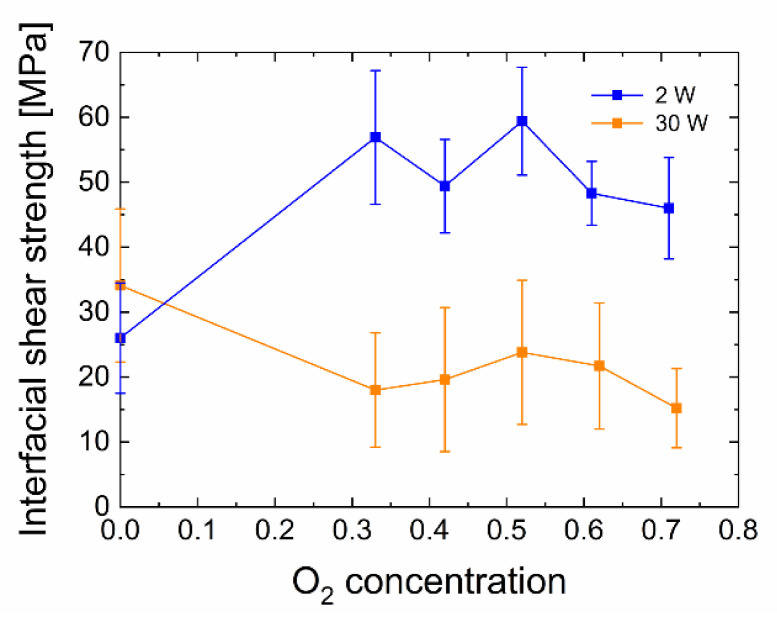
Influence of the oxygen concentration on interfacial shear strength (IFSS) values for two different powers.

Subsequently, with the knowledge of other quantities, namely the fibre radius and thin disk thickness, the IFSS for both sets (2 W and 30 W) was determined using Equation (2). The pushed-out fibres were inspected by optical microscopy and scanning electron microscopy (SEM) after performing the test; one of the SEM micrographs is shown in Figure 5. The resulting IFSS together with the standard deviations are shown in Figure 6.

For the a-CSiO:H interlayers from TVS/O_2_ mixtures with an oxygen content of 33–71% prepared at 2 W, we observed an increase in the interfacial shear strength of the composite samples in comparison to the a-CSi:H plasma nanocoatings from pure TVS. The IFSS values range from 46.0 to 59.4 MPa for the mixtures, in contrast to the plasma nanocoating of pure TVS that only reach 26.0 MPa. The plasma nanocoatings prepared at 30 W, for the sample from pure TVS, have an interfacial shear strength of 34.1 MPa. This again corresponds approximately to the 2 W plasma nanocoating from pure TVS. At higher oxygen concentrations, the IFSS decreases compared to the previous sample and the values range from 15.2 to 23.8 MPa. These trends can be explained as in the previous example of short-beam strength; see the previous section. In contrast to these values, however, the specified values of interfacial shear strength reach higher standard deviations. On average, they reach around 30% of the mean value. Such high values are probably caused by the necessary but relatively long polishing time of both surfaces in water, which may partially break the interface, even though the material is gradually removed during grinding and polishing. These higher standard deviations may also be affected to some extent by handling and manipulation of the sample, which must be done very carefully and precisely due to the dimensions of the thin disk in order to avoid bending and consequently mechanical stresses induced in the sample.

**Figure 7:**
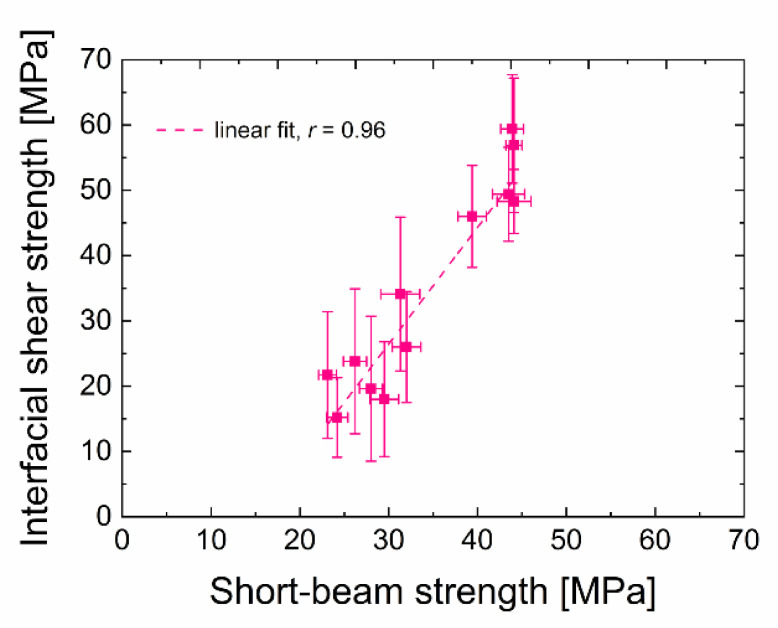
The relationship between IFSS and short-beam strength.

It is also essential to take into account that, during the micromechanical push-out test, the individual fibres are pushed out while the average value of the obtained measurements corresponds approximately to the average value of macromechanical testing by SBST, during which a large number of fibres is tested at once. In this way, the error of the SBST results is significantly reduced. The determined short-beam strength and interfacial shear strength correlate very well and achieve similar trends for both of these two sets of samples prepared from pure TVS and its mixtures with oxygen of different concentrations. The dependence of the IFSS on the short-beam strength is well-demonstrated in Figure 7. Linear regression was used to obtain a Pearson’s correlation coefficient r [46], indicating a correlation strength of 0.96 in this particular case. This suggests that these two obtained strengths are strongly correlated. However, it should be considered that the standard deviations are significant values of the IFSS, and in general, it is necessary to consider the small data set used. Therefore, this study requires more data to be validated. Nevertheless, we can conclude that the micromechanical test of the composite material and the macromechanical test provide similar results and that they are in good agreement.

Experimental data and model simulations suggest that the weak point of the polymer composite is the interface between the fibre surface and the plasma nanocoating used as a compatible interlayer [19]. Therefore, adhesion of the plasma nanocoating to the substrate is a key parameter. However, the results of both of these shear tests contradict the critical load trends. This may be due to a significant change in the mechanical properties of these tested plasma nanocoatings, such as the elastic modulus or the friction coefficient [23,24,47]. The correct measure of adhesion, therefore, may not be the critical load but the work of adhesion, as already discussed [20].

### 3.4. Adhesion of Plasma Nanocoatings in Terms of Work of Adhesion

Many nanoscratch testing parameters and settings were kept constant. However, the critical load is still affected by other intrinsic and extrinsic parameters. Therefore, using the critical load, we preferred to determine the work of adhesion using other material parameters. The work of adhesion was calculated using an equation derived from theoretical models [24,25,47,48]. This equation was subsequently modified to suit similar a-CSi:H and a-CSiO:H materials [42] as

(3) $$W_{\mathrm{adh}}=\frac{2\;t}{E}{\left(\frac{L_{c}\;\nu \;\mu_{c}}{\pi h_{c}\left(2R-h_{c}\right)}\right)}^{2},$$

where *h*_c_ is the track depth at the critical load *L*_c_ and *R* is the indenter tip radius. The track depth corresponds approximately to the thickness of the plasma nanocoating when reaching the *critical load*, i.e., the point of adhesion failure [42]. Thus, the track depth could be replaced by the determined plasma nanocoating thickness, as verified in previous study [20]. A fixed value of the Poisson’s ratio was used (*ν* = 0.35) [33], and the *L*_c_ values, the elastic modulus *E*, and the friction coefficient *μ* were determined experimentally.

From the aforementioned follows that the work of adhesion might be a more appropriate parameter to characterise the plasma nanocoating adhesion to the substrate because it eliminates not only the differences in the plasma nanocoating thickness but also the mechanical and tribological properties that strongly influence the critical normal load as a measure of adhesion.

#### 3.4.1. Mechanical and Tribological Properties of Plasma Nanocoatings

**Figure 8:**
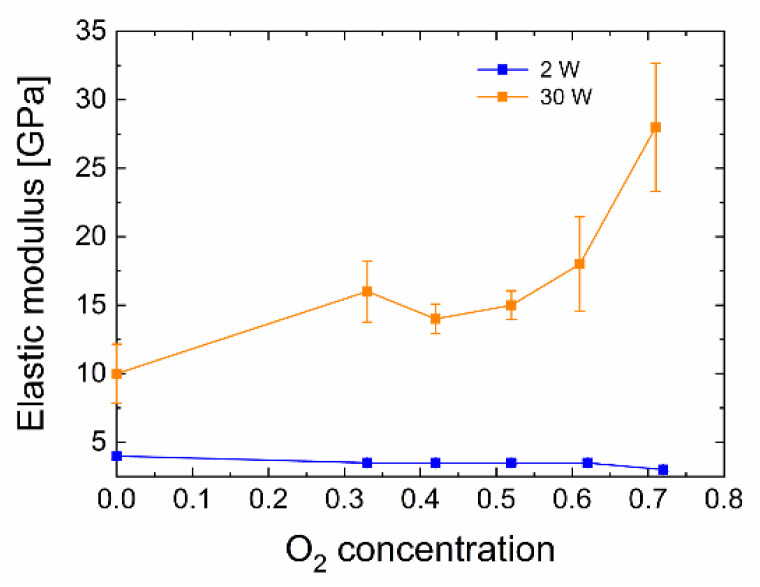
Influence of the oxygen concentration on the elastic modulus.

The values of the elastic modulus were determined for each of the prepared plasma nanocoatings. These results are shown in Figure 8. The plasma nanocoatings prepared at 30 W show a significant increase in elastic modulus from 10 to 28 GPa at increased oxygen concentration, especially in the 52 to 71% range. On the contrary, for the 2 W plasma nanocoating, with an increasing oxygen concentration in the mixture, we observed a slight decrease in the elastic modulus from 4.0 to 3.0 GPa, which is a value corresponding to the elastic modulus of the polyester resin (≈4 GPa [8]) used to prepare the composite samples.

**Figure 9:**
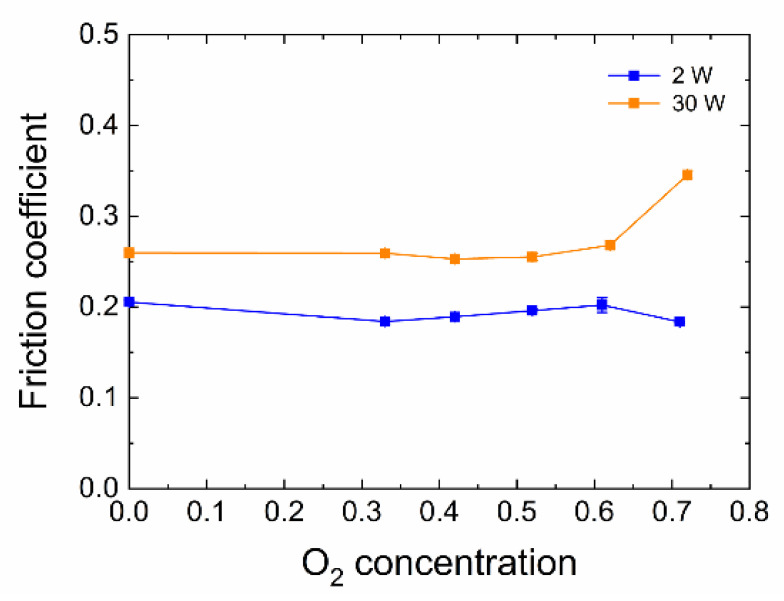
Graph of the friction coefficient vs. oxygen concentration.

In order to determine the work of adhesion, the friction coefficient was also observed; see Figure 9. The friction coefficient for 30 W samples was around 0.25; only the sample prepared from the TVS/O_2_ mixture containing 71% oxygen in the mixture reached a value of 0.34. For the 2 W series, the friction coefficient was around 0.19 and 0.20.

#### 3.4.2. Work of Adhesion

**Figure 10:**
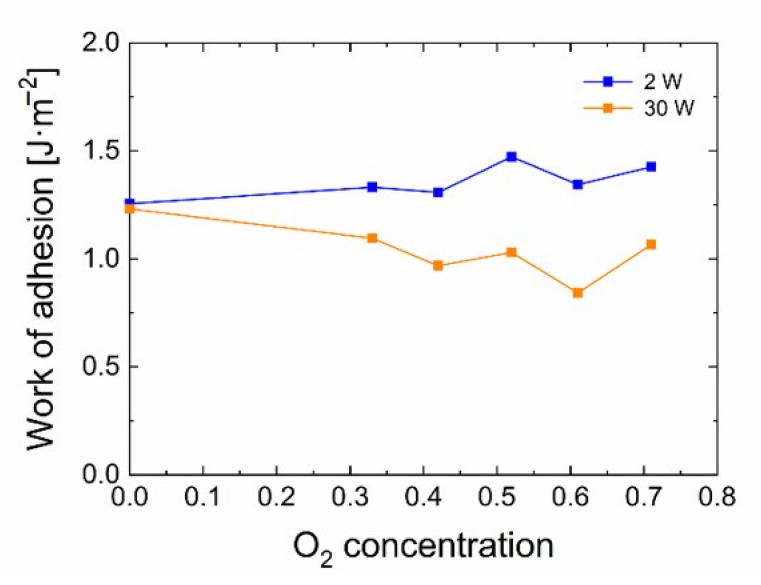
Calculated work of adhesion for the 2 W and 30 W sets of samples.

**Figure 11:**
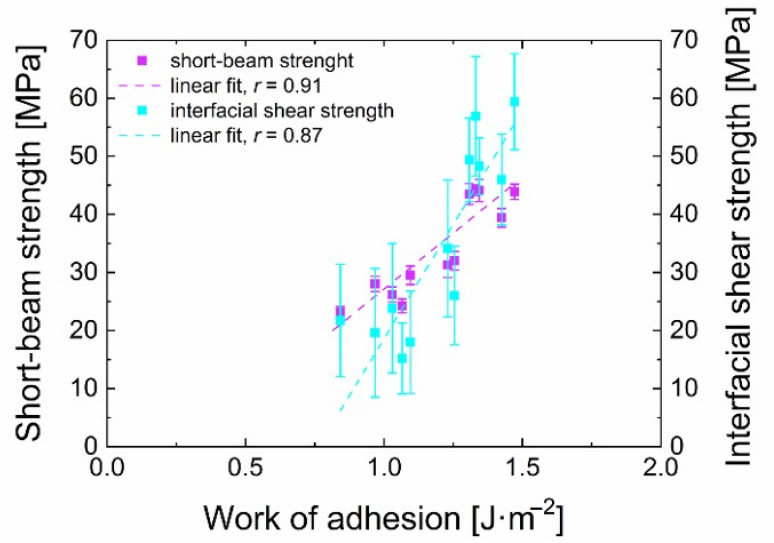
Relationships observed between short-beam strength/IFSS and the work of adhesion.

The obtained work of adhesion (Equation (3)) indicates in Figure 10, a similar trend as the graphs of the short-beam strength and the interfacial shear strength with regards to oxygen concentration for the two prepared series. These results suggest that the nanocoating adhesion is higher for 2 W samples than for 30 W samples. This corresponds very well to the results obtained by micromechanical and macromechanical tests of the GF/polyester composites. Nevertheless, it is necessary to be cautious because this quantity (work of adhesion) has a standard deviation usually between 10 to 15%, even though it occasionally reaches almost 30%. The short-beam strength and IFSS as a function of the work of adhesion were plotted and are shown in Figure 11. A relatively high value of the Pearson coefficient indicates a strong positive correlation between the short-beam strength and the work of adhesion (*r* = 0.91) and between the IFSS and the work of adhesion (*r* = 0.87) determined for plasma nanocoatings on silicon wafers.

It was again confirmed that the work of adhesion is a more suitable quantity for characterizing the adhesion of the plasma nanocoatings to the substrate in contrast to the critical normal load, especially for plasma nanocoatings with significant differences in mechanical or tribological properties, as shown by a previous study [20]. The observed values of the work of adhesion are in the same order of magnitude and correspond very well to the results obtained for the SiO_2_ and SiN_1_._3_ films on the polycarbonate substrate, as published by Rats et al. [43]. Similar values were also determined by other teams for the SiCN film on the porous SiOCH surface [49,50].

These results also suggest that determining the work of adhesion for a nanocoating on a silicon wafer may be a suitable method to select an interlayer with sufficient adhesion for the surface modification of GFs to reinforce a polymer composite.

## 4. Conclusions

The studied a-CSiH/a-CSiO:H plasma nanocoatings were prepared using 2 W effective power and 30 W RF (radio-frequency) power supplied to the plasma discharge with different oxygen concentrations (0–71% O_2_) in the TVS/O_2_ mixture. The critical loads for the 30 W samples were slightly higher than those for the 2 W samples. The elastic modulus of these plasma nanocoatings ranged from 3.0 to 28 GPa and the friction coefficient ranged from 0.19 to 0.34, whereas the samples deposited at 30 W always reached higher values of mechanical and tribological properties. However, the resulting trends in the work of adhesion were different in comparison with the critical normal load due to the elimination of the influence of these quantities such as elastic modulus, friction coefficient or plasma nanocoating thickness. The work of adhesion was determined to range from 0.8 to 1.5 mJ·m^−2^. These plasma nanocoatings were deposited on the GF bundles, which were used to prepare the GF/polyester composite short beams and subsequently thin disks. The short-beam strength of the composites was determined by the short beam shear test, and the IFSS was determined by the push-out test. Both of these tests indicated similar trends and were confirmed by their strong correlation. The composite samples with interlayers prepared from pure TVS reached similar values. All composites with 2 W plasma nanocoatings used as interlayers containing oxygen achieved significantly better results, which were on average twice as high as that for the interlayers deposited at 30 W. In comparison to the commercial sizing, a 13% higher short-beam strength was achieved.

Using macro- and micromechanical tests of composite beams, a strong correlation between both short-beam strength and IFSS, and the work of adhesion for plasma nanocoatings deposited on silicon wafers with a surface silicon dioxide overlayer was demonstrated. This strong correlation confirms the idea that the adhesion of plasma nanocoating to the glass substrate is a key parameter responsible for the shear properties of a GF/polyester composite. In other words, the adhesion of a plasma nanocoating used as an interlayer in the GF/polyester composite controls the shear properties of the composite material. In addition, the work of adhesion appears to be a more suitable parameter characterising nanocoating adhesion as opposed to the critical load due to elimination of the influence of the mechanical and tribological properties of the nanocoating.

The developed plasma nanocoatings, which control the shear strength of the polymer composite to a large extent through nanocoating adhesion, are very important for the design of the composite with regard to its use. The acquired knowledge will be the subject of further research. Knowledge of mechanical properties, such as elastic modulus, hardness and adhesion of individual nanocoatings, will lead to the design and synthesis of more complex interlayers, leading to high-performance composites without sharp interfaces using multilayers or gradient nanocoatings as interlayers.

## Figures and Tables

**Table 1:** Deposition condition.

**Frequency**	**13.56 MHz**
Base pressure	5 × 10^−4^ Pa
**Substrate pre-treatment**	
O_2_ gas pressure	5.3 Pa
O_2_ gas flow rate	10.0 sccm
RF power	30 W (flat substrates; 10 min) 100 W (bundle; 30 min)
**Plasma nanocoatings deposition**	
Process gas pressure	3.8 Pa
Oxygen fraction in TVS/O_2_ mixture	0–71%
Effective/RF power	2 W/30 W
Thickness for NS/NI testing	0.1 µm/1.0 µm
Plasma nanocoating thickness on GF bundles	>0.2 µm

## Data Availability

The data presented in this study are available on request from the corresponding author.
